# Comparison of One-Year Adverse Events Between First- and Second-Generation Bruton’s Tyrosine Kinase Inhibitors: A Retrospective Study

**DOI:** 10.7759/cureus.95858

**Published:** 2025-10-31

**Authors:** Ao Ito, Kaori Ito, Chisako Iriyama, Naoe Goto, Yoshihiro Inamoto, Masataka Okamoto, Yuichi Hirose, Misaki Morisaku, Shigeki Yamada, Nobuki Hayakawa, Akihiro Tomita

**Affiliations:** 1 Faculty of Pharmacy, Meijo University, Nagoya, JPN; 2 Department of Hematology, Fujita Health University School of Medicine, Toyoake, JPN; 3 Department of Blood and Marrow Transplantation and Cellular Therapy, Fujita Health University School of Medicine, Toyoake, JPN; 4 Department of Hematology and Oncology, Fujita Health University Okazaki Medical Center, Okazaki, JPN; 5 Department of Neurosurgery, Fujita Health University School of Medicine, Toyoake, JPN; 6 Department of Pharmacy, Fujita Health University Hospital, Toyoake, JPN

**Keywords:** acalabrutinib, adverse events, bleeding, bone marrow suppression, bruton’s tyrosine kinase inhibitors, cardiovascular toxicity, hematological malignancies, ibrutinib, infections

## Abstract

Background/Aim: Bruton’s tyrosine kinase inhibitors (BTKi) are important targeted agents for hematological malignancies. Second-generation BTKi are considered to have fewer off-target enzyme effects than first-generation agents; however, real-world comparative data on adverse events (AEs) remain limited. AEs of special interest with BTKi include bone marrow suppression, infection, hemorrhage, and cardiac-related events. This study aimed to investigate the frequency and severity of AEs of special interest associated with the three BTKi, namely, ibrutinib (IBR), tirabrutinib (TIR), and acalabrutinib (ACB), in real-world clinical practice.

Methods: We retrospectively investigated cytopenia and non-hematologic toxicities, including infections, bleeding, and cardiovascular AEs, for up to one year in patients who received BTKi at Fujita Health University Hospital and an affiliated hospital between March 2016 and March 2025 (IBR, n = 24; TIR, n = 24; ACB, n = 5). Data were collected from electronic medical records and graded according to the Common Terminology Criteria for Adverse Events, version 5.0.

Results: In the IBR group, the median age was 76 years (range, 76-81 years). Cytopenia, infections, bleeding, and cardiovascular AEs occurred in 21 (87.5%), nine (37.5%), eight (33.3%), and four (16.6%) patients, respectively. In the TIR group, the median age was 70 years (range, 64-76 years). Cytopenia, infections, and bleeding occurred in 17 (70.8%), seven (29.1%), and six (25.0%) patients, respectively. In the ACB group, the median age was 68 years (range, 52-75 years), and cytopenia was observed in four (80.0%) patients.

Conclusion: All BTKi agents were associated with bone marrow suppression, infection, and bleeding, whereas cardiac-related AEs occurred only with IBR. Several Grade 3 or higher events were identified, underscoring the need for careful monitoring of patients receiving BTKi in clinical practice.

## Introduction

Bruton’s tyrosine kinase (BTK) belongs to the TEC family of non-receptor tyrosine kinases and is widely expressed in multiple cell types, including B cells, mast cells, and neutrophils. Upon signaling through the B-cell receptor, complex signaling cascades are initiated, recruiting BTK to the cell membrane and activating other kinases. This process results in increased intracellular Ca^2+^ levels, activation of nuclear factor-kappa B, and stimulation of the mitogen-activated protein pathway [1].

Bruton’s tyrosine kinase inhibitors (BTKi) play an important role in the targeted treatment of hematological malignancies. These agents are believed to inhibit BTK kinase activity by either covalently binding to cysteine residue 481 or by competitively inhibiting the ATP-binding site of BTK, thereby suppressing the proliferation of B-cell tumors [2-7]. Currently, five BTKi agents are available in Japan [2-11].

Second-generation BTK inhibitors, including tirabrutinib (TIR) and acalabrutinib (ACB), are considered to have fewer off-target effects than the first-generation inhibitor, ibrutinib (IBR) [12,13]. However, many adverse events (AEs) in the real-world setting remain poorly characterized, and comparative studies between the two generations are still limited. This study aimed to investigate the frequency and severity of AEs of special interest for three drugs-IBR, TIR, and ACB-in real-world clinical practice.

## Materials and methods

Study design, patients, and data collection

We used a retrospective cohort study. Patients who received BTKi therapy (IBR, TIR, or ACB) at Fujita Health University Hospital and Fujita Health University Okazaki Medical Center between March 2016 and March 2025 were enrolled in this retrospective study. Eligibility criteria were patients who received BTKi treatment for hematological malignancies between March 2016 and March 2025. Exclusion criteria included patients for whom disease information was unknown. The cut-off date for data collection was March 31, 2025. If subsequent treatment initiation, death, or transfer to another hospital occurred within one year of BTKi initiation, the observation period ended at the earlier event. All data were obtained from electronic medical records, with AEs determined based on the attending physician’s assessment.

Assessments

Patient characteristics included diagnosis, age, sex, recurrence status, prior treatment history, number of previous treatment lines, initial BTKi dose, presence or absence of dose reduction, and treatment duration. Safety was evaluated by documenting the frequency and severity of AEs, including those that required dose modification or treatment discontinuation. AEs were graded according to the National Cancer Institute Common Terminology Criteria for Adverse Events, version 5.0 [14]. The time to onset of each AE was also recorded.

AEs of special interest were defined as bone marrow suppression, infection, hemorrhage, and cardiac-related events. Cardiac-related events included arrhythmias, heart failure, valvular heart disease, and hypertension [15]. AEs were recorded for up to 1 year from the initiation of BTKi therapy, at the following time points: one, three, six, nine, and 12 months (± 2 weeks). The cut-off date for data collection was March 2025. If subsequent treatment initiation, death, or transfer to another hospital occurred within one year of BTKi initiation, the observation period ended at the earlier event. All data were obtained from electronic medical records, with AEs determined based on the attending physician’s assessment.

Statistical analysis

No imputation was performed for missing data. Data were summarized using descriptive statistics, including means, standard deviations, and medians for continuous variables, and proportions for categorical variables. Because the comparison was not valid for these groups, statistical analysis was not performed. 95% confidence intervals were calculated using the Clopper-Pearson method. All analyses were performed using the R statistical software package (The R Foundation for Statistical Computing, Vienna, Austria; version 4.3.1) [16]. All analyses were performed using the R statistical software package (The R Foundation for Statistical Computing, Vienna, Austria; version 4.3.1) [16].

Ethical consideration

The Fujita Health University School of Medicine Epidemiological and Clinical Research Ethics Committee approved this retrospective study (HM24-279). All procedures involving human participants were conducted in accordance with the ethical standards of the institutional and/or national research committee and with the principles outlined in the Declaration of Helsinki and its subsequent amendments or comparable ethical standards. As this was a retrospective study, there were inherent limitations in data collection. Patient tolerance levels were assessed daily as necessary, and information regarding the clinical condition was consistently gathered throughout the observation period.

## Results

Patient disposition and demographics

**Figure 1:**
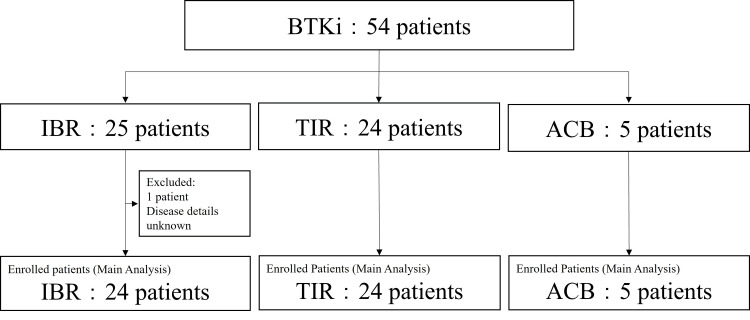
Patient flow chart

Overall, 54 patients were initially enrolled in this study, of whom 53 were included in the final analysis. Among these, 24, 24, and five patients received IBR, TIR, and ACB, respectively (Figure 1).

Table 1 summarizes patient characteristics. In the IBR group, 12 patients had chronic lymphocytic leukemia/small lymphocytic lymphoma (CLL/SLL), and 12 had mantle cell lymphoma (MCL). In the TIR group, eight patients had Waldenström’s macroglobulinemia/lymphoplasmacytic lymphoma, and 16 had primary central nervous system lymphoma. All five patients in the ACB group had CLL/SLL. The median age was >75, 70, and 68 years in the IBR, TIR, and ACB groups, respectively. Dose reduction rates were 45.8%, 29.1%, and 60% for the IBR, TIR, and ACB groups, respectively.

**Table 1:** Patient characteristics BTK: Bruton tyrosine kinase, IBR: Ibrutinib, TIR: Tirabrutinib, ACB: Acalabrutinib, CLL/SLL: Chronic lymphocytic leukemia/small lymphocytic lymphoma, MCL: Mantle cell lymphoma, WM/LPL: Waldenström's Macroglobulinemia/Lymphoplasmacytic lymphoma, PCNSL: Primary central nervous system lymphoma

BTK Inhibitor	IBR			TIR			ACB
	(n=24)			(n=24)			(n=5)
Disease	All	CLL/SLL	MCL	All	WM/LPL	PCNSL	CLL/SLL
Patients (n)	24	12	12	24	8	16	5
Age (median, range)(years)	76 (71–81)	76 (64–82)	76 (73–77)	70 (64–76)	70 (62–74)	69 (65–76)	68 (52–75)
Male (n, %)	13 (54.1)	7 (58.3)	6 (50.0)	14 (58.3)	7 (87.5)	7 (43.8)	3 (60.0)
Female (n, %)	11 (45.8)	5 (41.6)	6 (50.0)	10 (41.6)	1 (12.5)	9 (56.2)	2 (40.0)
Initial (%)	14 (58.3)	10 (83.3)	0 (0)	11 (45.8)	6 (75.0)	5 (31.2)	4 (80.0)
Relapse (%)	10 (41.6)	2 (16.6)	12 (100.0)	13 (54.1)	2 (25.0)	11 (68.7)	1 (20.0)
Pre-treatment history (yes, %)	15 (62.5)	3 (25.0)	12 (100.0)	19 (79.1)	3 (37.5)	16 (100.0)	2 (40.0)
Pre-treatment history (no, %)	9 (37.5)	9 (75.0)	0 (0)	5 (20.8)	5 (62.5)	0 (0)	3 (60.0)
Number of prior lines (median, range)	1 (0–2)	0 (0–0)	1 (1–2)	1 (1–1)	0 (0–1)	1 (1–1)	0 (0–1)
Starting dose (median, range)(mg)	350 (140–420)	210 (140–420)	420 (280–560)	480 (240–480)	280 (200–480)	480 (300–480)	200 (100–200)
Dose reduction (yes, %)	11 (45.8)	6 (50.0)	5 (41.6)	7 (29.1)	4 (50.0)	5 (31.2)	3 (60.0)
Dose reduction (no, %)	13 (54.1)	6 (50.0)	7 (58,3)	17 (70.8)	4 (50.0)	11 (68.7)	2 (40.0)
Duration of medication (median, range)(days)	293 (192–365)	316 (164–347)	282 (193–365)	124 (51–289)	310 (183–365)	97 (40–234)	164 (82–335)

AEs of special interest

Bone Marrow Suppression

The incidence and severity of cytopenia are presented in Table 2. In the IBR group, no cases of leukopenia were reported during the first year of treatment. Leukopenia was observed exclusively in the TIR group, and lymphopenia tended to occur more frequently with TIR than with IBR.

**Table 2:** Classification of the adverse events of cytopenia and their severities BTK: Bruton’s tyrosine kinase,  IBR: Ibrutinib, TIR: Tirabrutinib, ACB: Acalabrutinib, CLL/SLL: Chronic lymphocytic leukemia/small lymphocytic lymphoma, MCL: Mantle cell lymphoma, WM/LPL: Waldenström's Macroglobulinemia/Lymphoplasmacytic lymphoma, PCNSL: Primary central nervous system lymphoma, AEs: Adverse events, 95%CI: 95% confidence interval

BTK inhibitors	IBR				TIR				ACB	
	(n=24)				(n=24)				(n=5)	
Disease	CLL/SLL (n=12)		MCL (n=12)		WM/LPL (n=8)		PCNSL (n=16)		CLL/SLL	
Grade	All Grade	≥ Grade3	All Grade	≥ Grade3	All Grade	≥ Grade3	All Grade	≥ Grade3	All Grade	≥ Grade3
Cytopenia AEs, % (95%CI)	83.3 (0.52–0.97)	16.6 (0.020–0.48)	91.6 (0.61–0.99)	25.0 (0.054–0.57)	75.0 (0.34–0.96)	25.0 (0.031–0.65)	68.7 (0.41–0.88)	18.7 (0.040–0.45)	80.0 (0.28–0.99)	40.0 (0.052–0.85)
Leukepenia	0	0	0	0	0	0	5 (31.3)	1 (6.3)	1 (20.0)	0
Neutropenia	1 (8.3)	1 (8.3)	1 (8.3)	1 (8.3)	2 (25.0)	0	4 (25.0)	1 (6.3)	1 (20.0)	1 (20.0)
Lymphopenia	5 (41.6)	1 (8.3)	4 (33.3)	2 (16.6)	5 (62.5)	2 (25.0)	10 (62.5)	2 (12.5)	3 (60.0)	2 (40.0)
Thrombocytopenia	8 (66.6)	0	10 (83.3)	1 (8.3)	2 (25.0)	0	4 (25.0)	0	3 (60.0)	0

Infection

**Figure 2:**
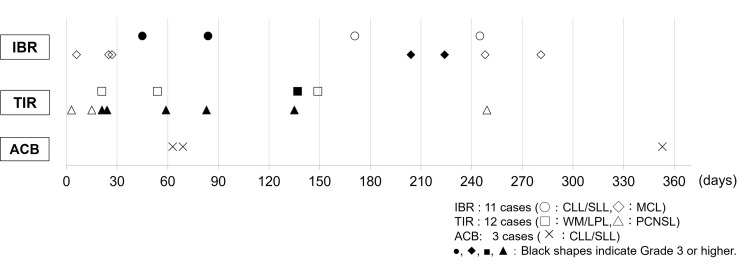
Timing of infectious complications

Table 3 outlines the classification and severity of each drug. Infection counts represent the number of patients affected, whereas the breakdown lists the number of cases for each infection type. No cases of infection were reported in the ACB group. Figure 2 illustrates the timing of infectious complications associated with each drug, showing that infections can occur both within the first year of BTKi initiation and over a longer period.

**Table 3:** Classification of infectious adverse events and their severities BTK: Bruton’s tyrosine kinase, IBR: Ibrutinib, TIR: Tirabrutinib, ACB: Acalabrutinib, CLL/SLL: Chronic lymphocytic leukemia/small lymphocytic lymphoma, MCL: Mantle cell lymphoma, WM/LPL: Waldenström's Macroglobulinemia/Lymphoplasmacytic lymphoma, PCNSL: Primary central nervous system lymphoma, AEs: Adverse events

BTK inhibitors	IBR				TIR				ACB	
	(n=24)				(n=24)				(n=5)	
Disease	CLL/SLL (n=12)		MCL (n=12)		WM/LPL (n=8)		PCNSL (n=16)		CLL/SLL (n=5)	
Grading	All	≥ Grade3	All	≥ Grade3	All	≥ Grade3	All	≥ Grade3	All	≥ Grade3
Infections AEs, patients, (%)	4 (33.3)	2 (16.6)	6 (50)	2 (16.6)	3 (37.5)	1(12.5)	4 (25.0)	2 (12.5)	3 (60.0)	0
Sepsis	1 (8.3)	1 (8.3)	0 (0)	0 (0)	0	0	0 (0)	0 (0)	0	0
Acute pneumonia	1 (8.3)	1 (8.3)	2 (16.6)	2 (16.6)	0	0	1 (6.3)	0	0	0
Cellulitis	0	0	1 (8.3)	1 (8.3)	0	0	0	0	0	0
COVID-19	0	0	2 (16.6)	0	2 (25.0)	0	1 (6.3)	0	1 (20.0)	0
Pharyngitis	0	0	0	0	0	0	0	0	1 (20.0)	0
Pneumocystis pneumonia	0	0	0	0	0	0	1 (6.3)	1 (6.3)	0	0
Cryptococcus meningitis	0	0	0	0	1 (12.5)	1 (12.5)	0	0	0	0
Herpes zoster viral encephalitis	0	0	0	0	0	0	1 (6.3)	1 (6.3)	0	0
Herpes zoster	1 (8.3)	0	0	0	0	0	0	0	0	0
Upper respiratory tract infection	0	0	1 (8.3)	0	1 (12.5)	0	0	0	1 (20.0)	0
Infectious rhinitis	1 (8.3)	0	0	0	0	0	0	0	0	0
Urinary tract infection	0	0	0	0	0	0	3 (18.8)	3 (18.8)	0	0
Tinea pedis	0	0	1 (8.3)	0	0	0	0	0	0	0
Tinea faciei	0	0	1 (8.3)	0	0	0	0	0	0	0
Tinea unguium	0	0	0	0	0	0	1 (6.3)	0	0	0

Bleeding

**Figure 3:**
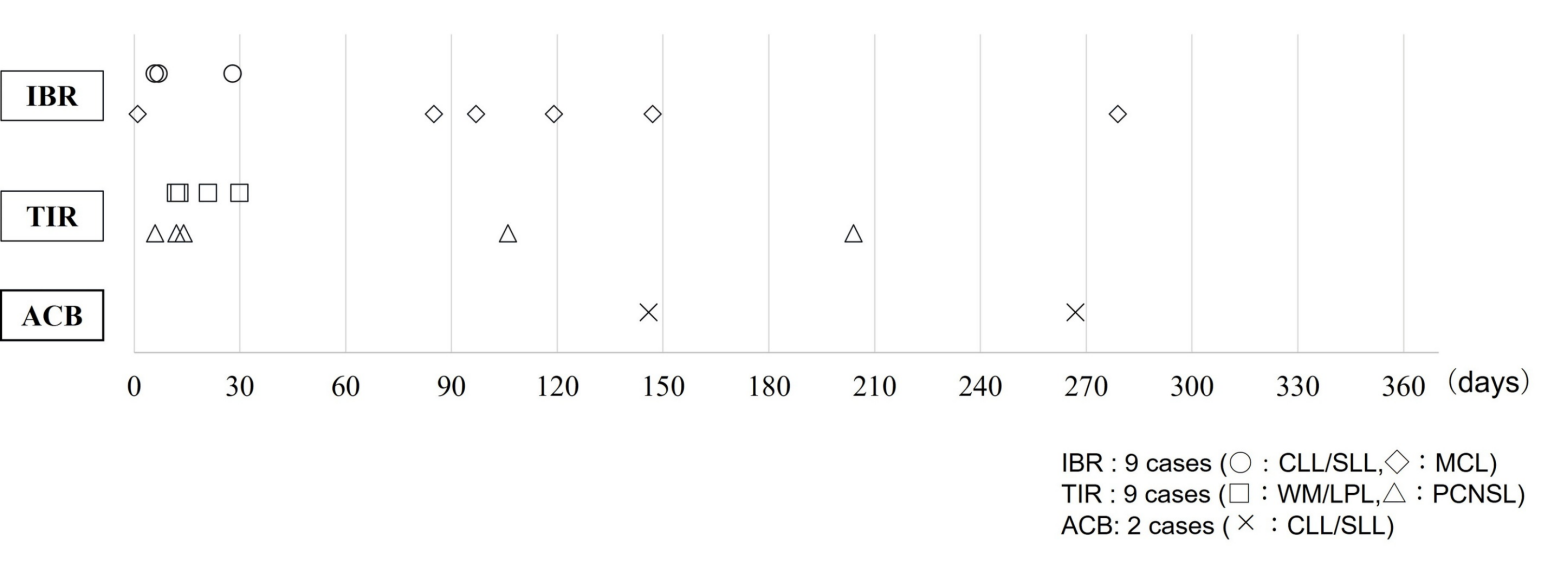
Timing of bleeding complications

The classification and severity of bleeding AEs are presented in Table 4. The total number of patients with bleeding complications is shown alongside the number of cases for each specific symptom. No bleeding events were reported in the ACB group. As illustrated in Figure 3, most bleeding symptoms occurred within four months of BTKi initiation; however, some symptoms developed beyond this period.

**Table 4:** Classification of bleeding and its severity BTK: Bruton’s tyrosine kinase, IBR: Ibrutinib, TIR: Tirabrutinib, ACB: Acalabrutinib, CLL/SLL: Chronic lymphocytic leukemia/small lymphocytic lymphoma, MCL: Mantle cell lymphoma, WM/LPL: Waldenström's Macroglobulinemia/Lymphoplasmacytic lymphoma, PCNSL: Primary central nervous system lymphoma, AEs: Adverse events

BTK inhibitors	IBR				TIR				ACB	
	(n=24)				(n=24)				(n=5)	
Disease	CLL/SLL (n=12)		MCL (n=12)		WM/LPL (n=8)		PCNSL (n=16)		CLL/SLL (n=5)	
Grading	All	≥ Grade 3	All	≥ Grade 3	All	≥ Grade 3	All	≥ Grade 3	All	≥ Grade 3
Bleeding AEs, Patients (%)	3 (25.0)	0	4 (33.3)	0	2 (25.0)	0	4 (25.0)	0	2 (40.0)	0
Petechiae	2 (16.7)	0	0	0	0	0	0	0	0	0
Subcutaneous bleeding	0	0	1 (8.3)	0	0	0	3 (18.7)	0	1 (20.0)	0
Purpura	0	0	1 (8.3)	0	0	0	1 (6.3)	0	1 (20.0)	0
Nosebleeds	0	0	2 (16.7)	0	3 (37.5)	0	0	0	0	0
Bleeding gums	1 (8.3)	0	1 (8.3)	0	1 (12.5)	0	0	0	0	0
Bloody stool	0	0	1 (8.3)	0	0	0	0	0	0	0
Hematuria	0	0	0	0	0	0	1 (6.2)	0	0	0

Cardiovascular AEs

Cardiovascular AEs were observed exclusively in the IBR group. One case of heart failure occurred in the CLL/SLL group, whereas two cases of arrhythmia and one case of hypertension were reported in the MCL subgroup. Of these, only the hypertension event was classified as Grade 3 or higher; the affected patients had a pre-existing history of hypertension. One case of arrhythmia developed more than 300 days after IBR initiation.

## Discussion

In this study, we compared the one-year AE profiles of the first-generation BTKi, IBR, with those of second-generation BTKi, TIR, and ACB, in real-world clinical practice, and described both the severity and timing of these events. Although data on such outcomes in real-world settings remain limited, we believe our findings provide meaningful insights for clinical practice. AEs such as cytopenia were observed with all BTKi agents; however, lymphopenia occurred more frequently with TIR than reported in a domestic phase II trial [17], warranting caution. Differences between our findings and those of domestic clinical trials may be attributable to variations in patient backgrounds and survey periods.

For IBR, consistent with previous reports [18], infections occurred even more than one year after treatment initiation, indicating the need for long-term vigilance. In contrast, the observation period for TIR was shorter than that for IBR, precluding a direct comparison between the two agents. A post-marketing multicenter study of IBR in MCL reported that 10.1% of patients experienced bleeding, with 2.4% experiencing Grade 3 or higher bleeding [19]. In our MCL cohort, bleeding occurred in 33.3% of patients, higher than previously reported, although no Grade 3 or higher events were observed. Prior studies [18] have shown that among anticoagulants or antiplatelet agents (19.4% of the safety analysis set), nine developed bleeding events (18.8%), whereas 16 bleeding events occurred in patients not receiving such agents (8.0%). In this study, concomitant use of anticoagulants or antiplatelet agents could not be evaluated, which may have influenced these results.

In the present study, cardiac-related AEs were observed only in patients receiving IBR. As previously reported [20], these events were also noted in patients with observation periods exceeding one year, underscoring the need for caution. Cardiac toxicity associated with IBR may be attributable to off-target effects absent in second-generation BTKi [21,22]. However, because the median treatment duration for TIR and ACB was shorter than that for IBR in our cohort, inter-drug differences should be interpreted carefully. Further investigation, including long-term follow-up beyond one year, is warranted.

This study had some limitations. First, the retrospective nature of the study may have introduced selection bias, as data were collected from patients who had already sought medical attention. Second, it was a single-center study with a limited sample size, making it difficult to identify factors contributing to AEs through multivariable analyses. Third, the inclusion of patients who meet the exclusion criteria for clinical trials necessitates cautious interpretation of our findings.

## Conclusions

This investigated the frequency and severity of AEs related to IBR, TIR, and ACB in real-world clinical practice. Bone marrow suppression, infection, and bleeding were common AEs observed with all three BTKi agents. However, cardiac-related AEs occurred only with IBR. Some AEs were of Grade 3 or higher, emphasizing the need for careful monitoring when administering BTKi in clinical practice. Therefore, further studies are warranted to refine monitoring protocols and improve the safety of BTKi therapy.
